# Effect of transcranial direct current stimulation for patients with disorders of consciousness: A systematic review and meta-analysis

**DOI:** 10.3389/fnins.2022.1081278

**Published:** 2023-01-23

**Authors:** Hui Ma, Kehong Zhao, Chengsen Jia, Jiuhong You, Mei Zhou, Tingting Wang, Cheng Huang

**Affiliations:** ^1^Department of Rehabilitation Medicine Center, West China Hospital, Sichuan University, Chengdu, Sichuan, China; ^2^School of Rehabilitation Medicine, West China School of Medicine, Sichuan University, Chengdu, Sichuan, China; ^3^Key Laboratory of Rehabilitation Medicine in Sichuan Province, Chengdu, Sichuan, China

**Keywords:** disorders of consciousness, transcranial direct current stimulation, meta-analysis, systematic review, coma recovery scale-revised

## Abstract

**Introduction:**

Transcranial direct current stimulation (tDCS) could potentially facilitate consciousness improvement in patients with disorders of consciousness (DOC). The aim of this study was to investigate the therapeutic efficacy of tDCS on consciousness recovery for patients with DOC.

**Methods:**

Eight databases were systematically searched from their inception to June 2022. Quality of included studies were assessed using PEDro score and Cochrane’s risk of bias assessment. All statistical analyses were performed using RevMan software. Seventeen studies with 618 patients were identified eligible for this study, and fifteen studies with sufficient data were pooled in the meta-analysis.

**Results:**

The results of meta-analysis showed a significant effect on increasing GCS scores (MD = 1.73; 95% CI, 1.28–2.18; *P* < 0.01) and CRS-R scores (MD = 1.28; 95% CI = 0.56–2.00; *P* < 0.01) in favor of the real stimulation group as compared to sham. The results of subgroup analysis demonstrated that only more than 20 sessions of stimulation could significantly enhance the improvement of GCS scores and the CRS-R scores. Moreover, the effect of tDCS on CRS-R score improvement was predominant in patients with minimal conscious state (MCS) (MD = 1.84; 95% CI = 0.74–2.93; *P* < 0.01).

**Conclusion:**

Anodal tDCS with sufficient stimulation doses appears to be an effective approach for patients with MCS, in terms of CRS-R scores.

**Systematic review registration:**

https://www.crd.york.ac.uk/PROSPERO/, identifier CRD42022336958.

## Introduction

A disorder of consciousness (DOC) is a state of medical condition that inhibit consciousness due to primary or secondary substantial brain injuries (Eapen et al., 2017). Conscious behavior requires two main components: adequate arousal and awareness of content. Disruption of one or both of these components could result in DOC (Bernat, 2006). DOC can be categorized into different types: coma, in which a patient is in deep state of prolonged consciousness, and fails to respond normally to internal or external stimulations; unresponsive wakefulness syndrome (UWS), which is previously known as vegetative state (VS), where a patient has sleep-wake cycle, but lacks awareness; minimal conscious state (MCS), where the patient has intermittent periods of awareness and wakefulness (Giacino et al., 2018). At a conservative estimate, about 5/100,000 people will enter a prolonged DOC from acute onset and progressive brain damage, and the incidence rate of DOC is growing, as the development of neurocritical care (Wade, 2018). As patients with DOC cannot participate in physical therapy actively, most of them have sever medical complications, including respiratory system disorders, skeletal muscle system disorders, endocrine and metabolic abnormalities, urinary system infection, autonomic nerve disorder, deep vein thrombosis and others, which would hinder the recovery process (Choi et al., 2008; Estraneo et al., 2018). Therefore, DOC patients place great financial strain on medical structures due to prolonged intensive care (Laureys and Schiff, 2012).

A lot of crucial work has been done on the accurate diagnosis of patients with DOC, which can lead to important medical decisions, such as withdrawal of life-sustaining care (Giacino et al., 2014; Boly et al., 2017). Nevertheless, no diagnostic assessment procedure had moderate or strong evidence for use in DOC (Giacino et al., 2018). Although neuroimaging and electrophysiologic procedures, including EMG, EEG, fMRI, and PET, are evolving as potential components of the DOC clinical assessment, there were insufficient evidentiary support to include them in formal diagnostic criteria or routine clinical care (Owen and Coleman, 2008; Schnakers et al., 2008). According to the American congress of rehabilitation medicine, the Coma Recovery Scale-Revised (CRS-R) with high sensitivity ranked the top-rated neurobehavioral rating scale for clinical assessment of patients with DOC (Seel et al., 2010). The CRS-R consists of 23 items comprised of six subscales designed to assess audition, receptive and expressive language, communication ability, visuoperception, motor functions and arousal level, including reflex behaviors and cognitively mediated behaviors (Annen et al., 2019). A CRS-R total score of 10 has 100% specificity for UWS, although also a false negative diagnostic error rate of 22% (Bodien et al., 2016). Therefore, most studies associated to DOC always selected CRS-R as an outcome measure or as a covariate in neuroimaging and neurophysiological analyses (Zhang et al., 2017; Feng et al., 2020). Meanwhile, the Glasgow Coma Scale is another clinical scale used to reliably measure a patient’s level of consciousness, which is widely used by neurosurgeons and nurses in more than 80 countries (Teasdale et al., 2014). Despite there are many neuroimaging and neuroelectrophysiological examinations, neurological and behavioral assessment is still the primary approach to determine the DOC progression, because it is generally believed that the higher-level behaviors correspond to higher levels of neurological functioning, as well as the ability to demonstrate lower-level behaviors or the disappearance of pathological behaviors as sign of recovery.

The neural mechanisms of DOC are complex and still unclear (Edlow et al., 2021). The mesocircuit fronto-parietal model supported that frontal cortex, central thalamus, brain stem, striatum and globus pallidus intema play important roles in consciousness processing, which are also intervention targets for DOC (Thibaut et al., 2019b). However, the clinical management of patients with DOC remains challenging, and the therapeutic options for DOC are also limited (Thibaut et al., 2019b). According to the 2018 edition of the Practice Guidelines for consciousness Disorders in the United States, no treatment for DOC has sufficient evidence to prove its absolute effectiveness (Giacino et al., 2018). The therapeutic options include pharmacological and non-pharmacological interventions. For pharmacological interventions, only few and limited evidence supported that patients with prolonged DOC could benefit from amantadine and zolpidem (Giacino et al., 2012; Whyte et al., 2014). Non-pharmacological interventions are always neuromodulation techniques attempting to promote DOC recovery by modulating brain excitability, including invasive and non-invasive brain stimulations (NIBS). Invasive brain stimulation consists of deep brain stimulation (DBS) and vagus nerve stimulation (VNS). NIBS consists of transcranial direct current stimulation (tDCS), repeated transcranial magnetic stimulation (rTMS), transcutaneous VNS and low intensity focused ultrasound pulse. Unfortunately, the therapeutic effects of such neuromodulation techniques are inconsistent and limited (Bourdillon et al., 2019). DBS is an invasive stimulation with severe side effects possibly (Lemaire et al., 2018). Due to the stimulation targets and parameters of DBS are various and methodological limitations, the overall quality of evidence based on the results of previous studies was not high (Bourdillon et al., 2019). VNS is a less invasive stimulation alternative to DBS, but only one case investigated its therapeutic potential in patients with DOC (Corazzol et al., 2017). rTMS is a non-invasive neuromodulation technique which can trigger firing of action potentials, but can induce epilepsy potentially, however, the level of evidence supporting its therapeutic effects of patients with DOC is low (Lefaucheur et al., 2014). tDCS delivers a weak intensity and continuous current to modulate the neural resting state membrane potential polarization, which is widely used in psychiatric mental illness and post stroke dysfunction previously (Palm et al., 2016; Sehm, 2017). Compared with rTMS, tDCS is less possible to induce epilepsy and its therapeutic effects last more than a few minutes which could induce after-effects mediated by synaptic pathways (Kronberg et al., 2017). Moreover, the equipment of tDCS is inexpensive and implemented without site restrictions, which is more convenient to use at bedside or at home than rTMS. Since Thibaut et al. firstly published a sham-controlled randomized study on tDCS for patients with DOC in 2014, more researchers investigated the efficacy of tDCS for patients with DOC, however, due to the various stimulation parameters, the results were conflicting and controversial (Thibaut et al., 2014). A meta-analysis assessing the effects of NIBS in patients with DOC concluded that patients with MCS could benefit from tDCS, but no dose-session effect was found (Feng et al., 2020). The authors stated that additional high-quality studies were required to validate their findings. Some well-designed studies investigating the role of tDCS in patients with DOC were published recently (Chen et al., 2021; Guo et al., 2021; Li et al., 2021; Barra et al., 2022). Consequently, the present systematic review and meta-analysis aimed to integrate new evidence presented in recent years to evaluate the efficacy of tDCS for patients with DOC.

## Methods

The present systematic review and meta-analysis were performed and reported in line with the Preferred Reporting Items for Systematic Reviews and Meta-Analysis 2020 statement (PRISMA 2020), and *Cochrane Handbook for Systematic Reviews of Interventions* (Cumpston et al., 2019; Page et al., 2021). In addition, the present systematic review was registered in the International Prospective Register of Systematic Reviews (PROSPERO): CRD42022336958.

### Data sources and search strategies

We systematically searched for relevant articles available in both Chinese and English in electronic databases, including MEDLINE (*via* Ovid), Web of Science, Embase (*via* Ovid), CENTRAL (Cochrane library), Physiotherapy Evidence Database (PEDro), Chinese National Knowledge Infrastructure (CNKI), Wanfang Data and Weipu Database from their inception until June 2022. Search terms included key words associated with DOC, MCS, VS, and tDCS. The specific search strategy of all databases used are presented in Supplementary Digital Content 1. Furthermore, a manual screening of reference lists of the articles was performed to identify additional relevant studies. No ethical approval or patient consent was required because all analyses were based on previously published studies.

### Study selection

Endnote software was used to check for duplicated studies. Two investigators reviewed the studies independently and selected studies based on the predetermined criteria. All potentially relevant articles were retrieved from the databases for the assessment of their full text based on titles and abstracts. Studies that did not meet the inclusion criteria were excluded. Discrepancies between two reviewers were resolved through discussions with a third reviewer until a consensus was reached. The included studies were required to meet the following criteria: (1) studies were RCTs in either parallel or cross-over design published in English or Chinese, (2) studies were recruited adult participants with DOC, (3) intervention treatments were tDCS and sham stimulation as the control, and (4) with regard to outcome measures, studies used CRS-R or GCS as outcome measure for the recovery of DOC. Studies meeting any of these criteria were excluded: (1) studies published in dissertations, conference abstracts, or other types without peer-review; (2) non-randomized controlled trials or outcome measures without GCS or CRS-R scores; (3) studies published in neither English nor Chinese.

### Data extraction and quality assessment

Two reviewers independently extracted relevant data onto a pre-developed data extraction sheet, and disagreements were adjudicated by a third reviewer. The data extracted from selected studies included basic information (first author, year of publication), study design, demographic characteristics of patients (sample size, patient diagnosis), details of interventions applied to the experimental and control groups (stimulation protocol, brain target, and stimulation dose), relevant outcome measures.

Eligible articles were scrutinized for methodological quality by two independent reviewers using PEDro scale. The PEDro scale comprises 11 items with a total score ranging from 0 to 10 (except for item 1). The methodological quality of studies scoring 9–10 was considered to be of “excellent” quality, studies scoring 6–8 were considered to be of “good” quality, studies scoring 4–5 were considered to be of “fair” quality, and studies scoring below 4 were considered to be of “poor” quality (Foley et al., 2003). Discrepancies between two reviewers were resolved through discussions with a third reviewer. Additionally, risk of bias assessments were performed using the criteria described in the *Cochrane Handbook for Systematic Reviews of Interventions* (Cumpston et al., 2019). The evaluation entries included the following aspects: random sequence generation, allocation concealment, masking, incomplete outcome data, and selective outcome reporting among others. The included articles were evaluated as “low risk,” “high risk,” or “unclear risk.” Quality assessment was not used as a selection or exclusion criterion.

### Data synthesis and analysis

The results of all included studies were pooled using standard meta-analytic methods to estimate the effect of tDCS for the recovery of DOC. Based on the nature of extracted data, we assessed the mean differences (MDs) and 95% confidence intervals (CIs) for continuous outcomes. A *P*-value < 0.05 (two-sided) was considered statistically significant in the estimation of effects. Statistical heterogeneity was evaluated using chi-square test and *I*^2^ statistic. *P*-value < 0.05 or *I*^2^ value > 40% was considered high heterogeneity. A fixed-effects model was used when *P*-value was > 0.05; otherwise, a random-effects model was used. Sensitivity analyses were performed by excluding each study from the analysis when heterogeneity was detected, and the subgroup analyses were performed based on the different stimulation protocols, stimulation doses or patient diagnoses. Publication bias was not assessed due to the limited number of included studies. All statistical analyses were performed using RevMan software (Version 5.3; Cochrane Collaboration, Copenhagen, Denmark).

## Results

### Search results

**FIGURE 1:**
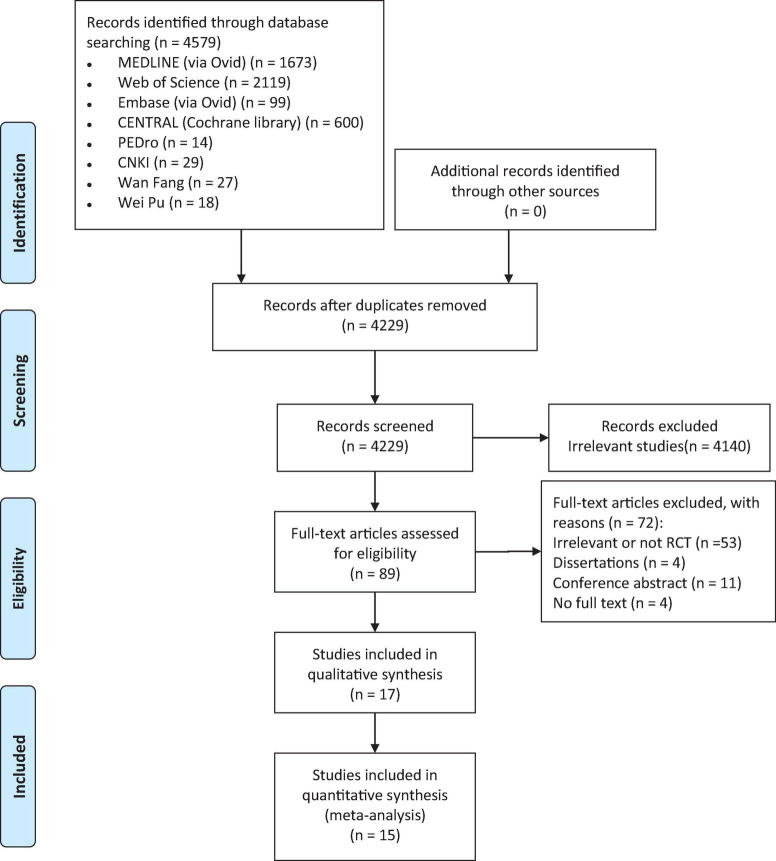
PRISMA flow diagram.

The initial electronic search resulted in a total of 4,579 studies, of which 4,229 unique articles were retrieved after duplicates were removed. After screening the titles, abstracts, and full text of the articles based on the inclusion and exclusion criteria, 17 studies (Thibaut et al., 2014, 2017, 2019a; Estraneo et al., 2017; Huang et al., 2017; Zhang et al., 2017, 2020; Chi et al., 2018; Martens et al., 2018, 2019, 2020; Cavinato et al., 2019; Wu et al., 2019; Chen et al., 2021; Guo et al., 2021; Li et al., 2021; Barra et al., 2022) with a total of 618 participants with DOC were identified as eligible for the systematic review. Two studies did not report enough data for calculating effect size and therefore were excluded from the meta-analysis (Cavinato et al., 2019; Thibaut et al., 2019a). Finally, 15 studies with 580 DOC patients were included in the quantitative synthesis (Thibaut et al., 2014, 2017; Estraneo et al., 2017; Huang et al., 2017; Zhang et al., 2017, 2020; Chi et al., 2018; Martens et al., 2018, 2019, 2020; Wu et al., 2019; Chen et al., 2021; Guo et al., 2021; Li et al., 2021; Barra et al., 2022). The details of the search process are shown in Figure 1.

### Description of studies

The studies included in this systematic review were published between 2014 and 2022. Five of them were published in Chinese (Chi et al., 2018; Zhang et al., 2020; Chen et al., 2021; Guo et al., 2021; Li et al., 2021) and 12 of them were published in English (Thibaut et al., 2014, 2017, 2019a; Estraneo et al., 2017; Huang et al., 2017; Zhang et al., 2017; Martens et al., 2018, 2019, 2020; Cavinato et al., 2019; Wu et al., 2019; Barra et al., 2022). The sample size ranged from 10 to 113 participants. The characteristics of included studies, including study design, patient diagnosis, details of intervention, and outcome measures, were summarized in Table 1.

**TABLE 1:** Characteristics of included studies in this review.

References	Study design	Participants	Intervention	Brain target	Duration	Outcome
Barra et al. (2022)	Double blind, randomized, cross-over	12 DOC	Group 1: 6–10 Hz tPCS with a biphasic current of 2 mA peak to peak Group 2: maximum of 2 mA anodal tDCS Group 3: sham stimulation 5-day washout	Bi-mastoid LDLPFC (F3)	tDCS: 20 min for one session tPCS: 20 min for one session	EEG CRS-R Side effect
Cavinato et al. (2019)	Double blind, randomized, cross-over	24 DOC (12 MCS, 12 UWS)	EG: 2 mA anodal tDCS CG: sham tDCS 10-day washout	LDLPFC (F3)	20 min per session, 1 session per day, 5 days per week for 2 consecutive weeks	EEG CRS-R WNSSP
Chen et al. (2021)	Randomized, parallel group	52 DOC	EG: 2 mA anodal tDCS paired with 50 Hz and 200 μs MNES CG: conventional therapy only	LDLPFC (F3) Right median nerve	tDCS: 20 min per session, 1 session per day, 6 days per week for 4 consecutive weeks MNSE: 30 min per session, 2 sessions per day, 6 days per week for 4 consecutive weeks	GCS GOS DRS BAEP USEP
Chi et al. (2018)	Randomized, parallel group	38 DOC	EG: 2 mA anodal tDCS paired with conventional therapy CG: conventional therapy only	LDLPFC (F3)	20 min per session, 1 session per day, 6 days per week for 20 sessions	BAEP USEP EEG GCS PVS
Estraneo et al. (2017)	Double blind, randomized, cross-over	13 DOC (7 VS, 6 MCS)	EG: 2 mA anodal tDCS CG: sham tDCS 1-week washout	LDLPFC (F3)	20 min per session, 1 session per day for five sessions	CRS-R EEG
Guo et al. (2021)	Randomized, parallel group	113 DOC	EG: 1.4 mA anodal tDCS paired with perceptual level arousal intervention CG: perceptual level arousal intervention only	LDLPFC (F3)	20 min per session, 1 session per day, 6 days per week for 4 consecutive weeks	CRS-R GCS DFS EEG Latency of evoked action potential
Huang et al. (2017)	Double blind, randomized, cross-over	37 MCS	EG: 2 mA anodal tDCS CG: sham tDCS 5-day washout	LDLPFC (F3)	20 min per session, 1 session per day for five sessions	CRS-R
Li et al. (2021)	Randomized, parallel group	102 DOC	Group 1: 2 mA anodal tDCS paired with conventional therapy Group 2: 60 Hz and 250 μs MNES paired with conventional therapy Group 3: tDCS and MNES paired with conventional therapy	LDLPFC (F3) Right median nerve	20 min per session, 1 session per day, 6 days per week for 8 consecutive weeks	Somatosensory evoked potential GCS
Martens et al. (2018)	Double blind, randomized, cross-over	27 DOC	EG: 2 mA anodal tDCS paired with conventional therapy CG: sham tDCS paired with conventional therapy 8-week washout	LDLPFC (F3)	20 min per session, 1 session per day, 5 days per week for 4 consecutive weeks	Adverse events CRS-R
Martens et al. (2019)	Double blind, randomized, cross-over	10 DOC (4 UWS, 6MCS)	EG: 2 mA anodal tDCS CG: sham tDCS 24-h washout	Primary motor cortex (C3-C4)	20 min for one session	CRS-R
Martens et al. (2020)	Double blind, randomized, cross-over	46 DOC (17 UWS, 23 MCS, 6 EMCS)	EG: tDCS with 4 anodes and 4 cathodes, 1 mA per anode CG: sham tDCS 2–6-day washout	Anodes placed on F3, F4, CP5 and CP6	20 min for one session	CRS-R EEG
Thibaut et al. (2014)	Double blind, randomized, cross-over	25 VS/UWS 30 MCS	EG: 2 mA anodal tDCS paired with conventional therapy CG: sham tDCS paired with conventional therapy 2-days washout	LDLPFC (F3)	20 min a single session	CRS-R
Thibaut et al. (2017)	Double blind, randomized, cross-over	16 MCS	EG: 2 mA anodal tDCS paired with conventional therapy CG: sham tDCS paired with conventional therapy 1-week washout	LDLPFC (F3)	20 min per session, 1 session per day for 5 consecutive days;	CRS-R
Thibaut et al. (2019a)	Double blind, randomized, cross-over	14 DOC	EG: 1 mA anodal tDCS paired with conventional therapy CG: sham tDCS paired with conventional therapy 2-days washout	LDLPFC (F3) RDLPFC (F4)	20 min a single session	MAS CRS-R EEG
Wu et al. (2019)	Randomized, parallel group	15 DOC	Group 1: 2 mA anodal tDCS anode placed over the left DLPFC paired with conventional therapy Group 2: 2 mA anodal tDCS anode placed over the right DLPFC paired with conventional therapy Group 3: sham tDCS paired with conventional therapy	LDLPFC (F3) RDLPFC (F4)	20 min per session, 1 session per day, 10 working days (from Monday to Friday in two consecutive weeks).	CRS-R GOS-E EEG
Zhang et al. (2017)	Double blind, randomized, parallel	26 DOC (11VS, 15MCS)	EG: 2 mA anodal tDCS paired with conventional therapy CG: sham tDCS paired with conventional therapy	LDLPFC (F3)	20 min per session, 2 session per day, 10 consecutive working days (from Monday to Friday).	CRS-R ERP
Zhang et al. (2020)	Double blind, randomized, parallel group	18 MCS	EG: 2 mA anodal tDCS paired with conventional therapy CG: sham tDCS paired with conventional therapy	LDLPFC (F3)	20 min per session, 2 sessions per day for 10 consecutive working days	CRS-R ERP

CG, control group; DIT, diffusion tensor imaging; BAEP, brain stem auditory evoked potential; DRS, disability rating scale; EEG, electroencephalogram; EG, experimental group; EMCS, emerged from minimally conscious state; EMG, electromyography; ERP, event-related potentials; FOUR, full outline of unresponsiveness scale; GCS, Glasgow coma scale; GOS, Glasgow outcome scale; L/RDLPFC, left/right dorsolateral prefrontal cortex; MBI, modified Barthel index; MCS, minimally conscious state; PVS, persistent vegetative state; tDCS, transcranial direct current stimulation; tPCS, transcranial pulsed-current stimulation; USEP, upper limb somatosensory evoked potential; UWS, unresponsive wakefulness syndrome; WNSSP, western neurosensory stimulation profile.

All studies included in the current systematic review and meta-analysis satisfied specific inclusion and exclusion criteria. For study design, seven studies were randomized parallel design (Zhang et al., 2017, 2020; Chi et al., 2018; Wu et al., 2019; Chen et al., 2021; Guo et al., 2021; Li et al., 2021), and ten studies were randomized cross-over design (Thibaut et al., 2014, 2017, 2019a; Estraneo et al., 2017; Huang et al., 2017; Martens et al., 2018, 2019, 2020; Cavinato et al., 2019; Barra et al., 2022). All participants in the selected studies were diagnosed with different degrees of DOC. Nine studies distinguished between MCS and VS/UWS (Thibaut et al., 2014, 2017; Estraneo et al., 2017; Huang et al., 2017; Zhang et al., 2017, 2020; Cavinato et al., 2019; Martens et al., 2019, 2020), while the other eight studies did not (Chi et al., 2018; Martens et al., 2018; Thibaut et al., 2019a; Wu et al., 2019; Chen et al., 2021; Guo et al., 2021; Li et al., 2021; Barra et al., 2022). For intervention strategies, all experimental groups received anodal tDCS targeting F3, except one study with four anodal tDCS targeting F3, F4, CP5, and CP6 (Martens et al., 2020). For stimulation doses, the intervention period ranged from 1 day to 8 weeks. Five studies conducted a single session of tDCS totally (Thibaut et al., 2014, 2019a; Martens et al., 2019, 2020; Barra et al., 2022), and 12 studies conducted five or more sessions of tDCS totally (Estraneo et al., 2017; Huang et al., 2017; Thibaut et al., 2017; Zhang et al., 2017, 2020; Chi et al., 2018; Martens et al., 2018; Cavinato et al., 2019; Wu et al., 2019; Chen et al., 2021; Guo et al., 2021; Li et al., 2021). Outcomes were measured at baseline and at the end of the intervention. 14 studies used CRS-R to evaluate the DOC, four studies used GCS and one study used both scales to evaluate the DOC.

### Quality

**FIGURE 2:**
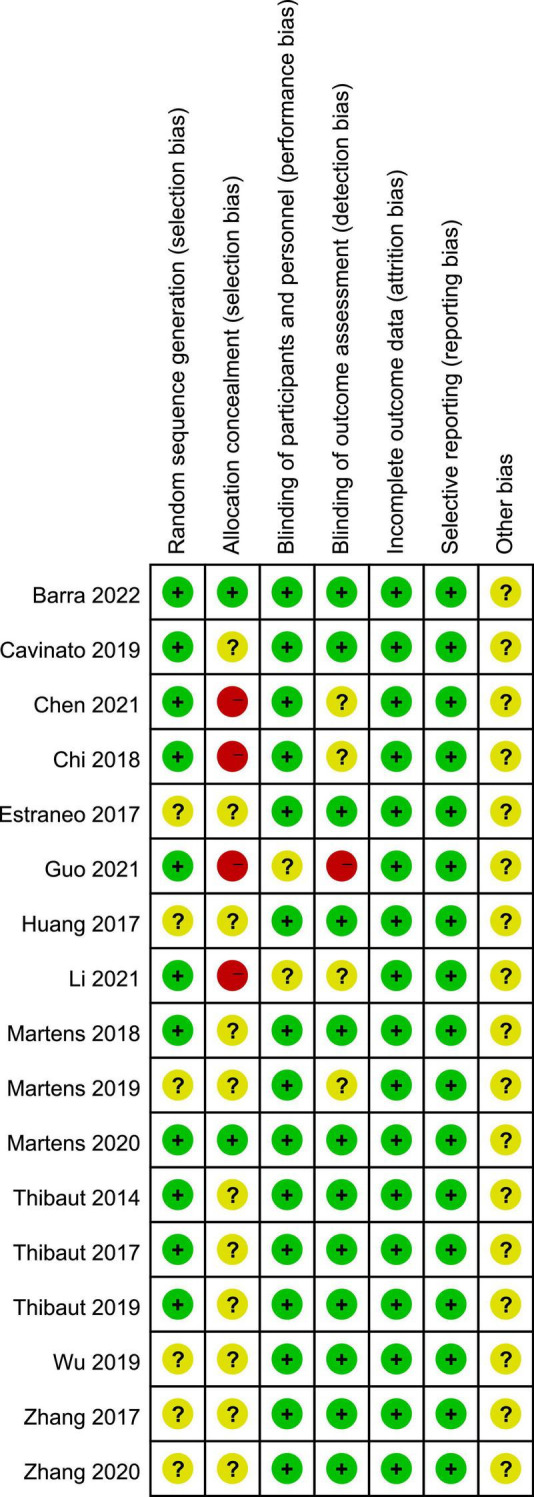
Risk of bias summary according to the Cochrane risk of bias tool: “-”, “+”, and “?” indicate high, low, and unclear risk of bias, respectively.

**FIGURE 3:**
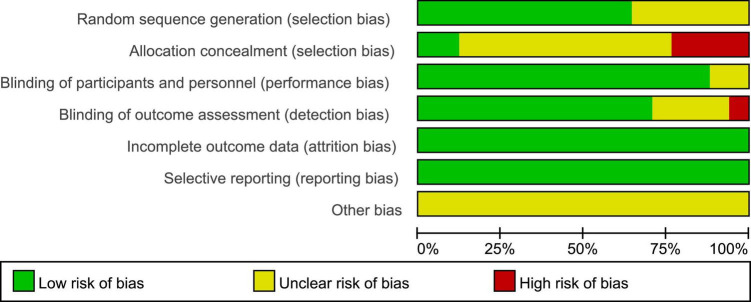
Risk of bias graph according to the Cochrane risk of bias tool.

PEDro scores of the included studies ranged from 6 to 9, with a mean score of 7.88, indicating a high methodological quality of our included studies. The methodological quality of six studies was considered to be of “excellent” quality (Thibaut et al., 2014, 2019a; Huang et al., 2017; Cavinato et al., 2019; Martens et al., 2020; Barra et al., 2022), while that of 11 studies was considered to be of “good” quality (Estraneo et al., 2017; Thibaut et al., 2017; Zhang et al., 2017, 2020; Chi et al., 2018; Martens et al., 2018, 2019; Wu et al., 2019; Chen et al., 2021; Guo et al., 2021; Li et al., 2021). A detailed evaluation of the PEDro scores is presented in Table 2. All included studies reported adequately with regard to their random sequence generation and baseline comparability. Unfortunately, no studies satisfied the concealed allocation criteria. Four studies did not satisfy the subject blinding (Chi et al., 2018; Chen et al., 2021; Guo et al., 2021; Li et al., 2021), six studies did not satisfy the therapist blinding (Zhang et al., 2017; Chi et al., 2018; Wu et al., 2019; Chen et al., 2021; Guo et al., 2021; Li et al., 2021), and six studies did not state assessor blinding (Chi et al., 2018; Martens et al., 2019; Zhang et al., 2020; Chen et al., 2021; Guo et al., 2021; Li et al., 2021). Risk of bias assessment of the studies included in the present systematic review and meta-analysis is illustrated in Figures 2, 3.

**TABLE 2:** PEDro assessment quality results of included studies.

References	Eligibility*	Random allocation	Concealed allocation	Baseline comparability	Blind subjects	Blind therapists	Blind assessors	Adequate follow-up	Intention-to-treat analysis	Between-group comparisons	Point estimates and variability	Total score (0–10)	Quality
Barra et al. (2022)	Yes	1	0	1	1	1	1	1	1	1	1	9	Excellent
Cavinato et al. (2019)	Yes	1	0	1	1	1	1	1	1	1	1	9	Excellent
Chen et al. (2021)	Yes	1	0	1	0	0	0	1	1	1	1	6	Good
Chi et al. (2018)	Yes	1	0	1	0	0	0	1	1	1	1	6	Good
Estraneo et al. (2017)	Yes	1	0	1	1	1	1	1	1	1	0	8	Good
Guo et al. (2021)	Yes	1	0	1	0	0	0	1	1	1	1	6	Good
Huang et al. (2017)	Yes	1	0	1	1	1	1	1	1	1	1	9	Excellent
Li et al. (2021)	YES	1	0	1	0	0	0	1	1	1	1	6	Good
Martens et al. (2018)	Yes	1	0	1	1	1	1	0	1	1	1	8	Good
Martens et al. (2019)	Yes	1	0	1	1	1	0	1	1	1	1	8	Good
Martens et al. (2020)	Yes	1	0	1	1	1	1	1	1	1	1	9	Excellent
Thibaut et al. (2014)	Yes	1	0	1	1	1	1	1	1	1	1	9	Excellent
Thibaut et al. (2017)	Yes	1	0	1	1	1	1	0	1	1	1	8	Good
Thibaut et al. (2019a)	Yes	1	0	1	1	1	1	1	1	1	1	9	Excellent
Wu et al. (2019)	Yes	1	0	1	1	0	1	1	1	1	1	8	Good
Zhang et al. (2017)	Yes	1	0	1	1	0	1	1	1	1	1	8	Good
Zhang et al. (2020)	Yes	1	0	1	1	1	0	1	1	1	1	8	Good

*Eligibility criteria is not included in the scoring of PEDro scale.

### Effect of intervention

#### Glasgow coma scale

**FIGURE 4:**
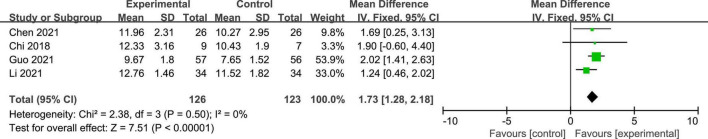
Meta-analysis of all studies on GCS scores in patients with DOC.

**FIGURE 5:**
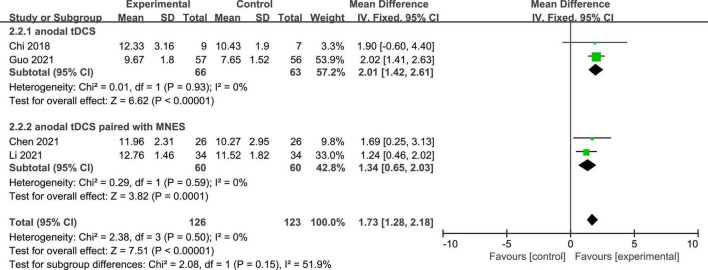
Subgroup analysis of stimulation protocol on GCS socres in patients with DOC.

**FIGURE 6:**
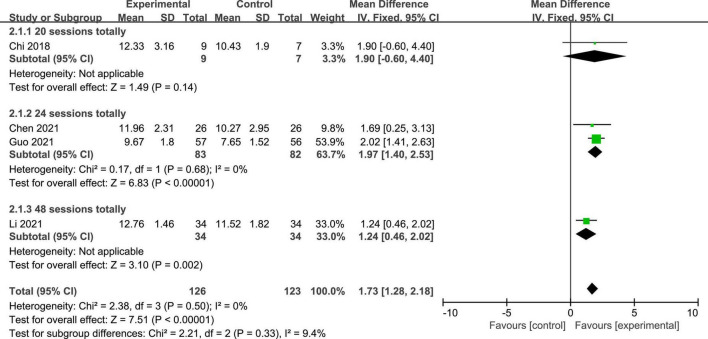
Subgroup analysis of stimulation doses on GCS scores in patients with DOC.

Four studies reported the GCS scores of patients with DOC. A fixed-effects model was used for the meta-analysis of GCS scores. The results of meta-analysis indicated that GCS increased significantly in favor of the intervention group (*MD* = 1.73; 95% CI, 1.28–2.18; *P* < 0.01; Figure 4). On the basis of subgroup analysis for stimulation protocol, two studies used anodal tDCS paired with median nerve electrical stimulation (MNES) and two studies used anodal tDCS, for intervention group. The results of meta-analysis showed that the GCS scores of both stimulation protocols increased significantly when compared to the control group (anodal tDCS paired with MNES: *MD* = 1.34; 95% CI = 0.65–2.03; *P* < 0.01; anodal tDCS: *MD* = 2.01; 95% CI = 1.42–2.61; *P* < 0.01; Figure 5). Furthermore, for the subgroup analysis of stimulation doses, on study conducted 20 sessions of stimulation totally (*MD* = 1.90; 95% CI = −0.60–4.40; *P* = 0.14), two studies conducted 24 sessions totally (*MD* = 1.97; 95% CI = 1.40–2.53; *P* < 0.01), and one study conducted 48 sessions of stimulation totally (*MD* = 1.24; 95% CI = 0.46–2.02; *P* < 0.01; Figure 6). No heterogeneity was detected among these studies in all above meta-analysis (*I*^2^ = 0%; *P* > 0.10). Publication bias was not assessed due to the limited number of included studies.

#### Coma recovery scale-revised

**FIGURE 7:**
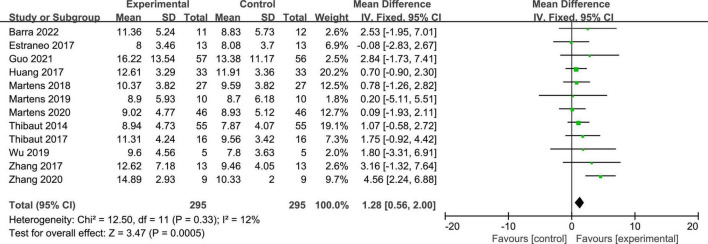
Meta-analysis of all studies on CRS-R scores in patients with DOC.

**FIGURE 8:**
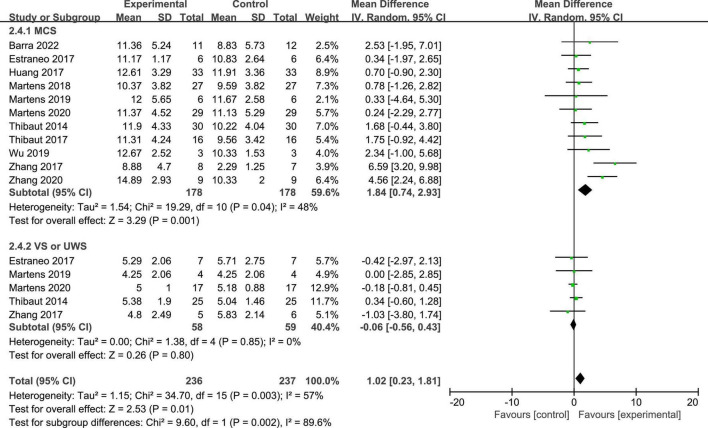
Subgroup analysis of patient diagnosis on GCS scores in patients with DOC.

**FIGURE 9:**
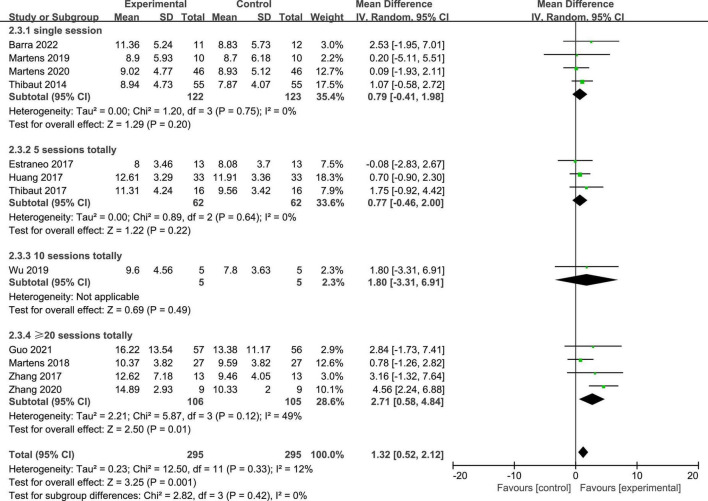
Subgroup analysis of stimulation doses on CRS-R scores in patients with DOC.

Twelve studies reported the CRS-R scores of patients with DOC. A fixed-effects model was used for the meta-analysis of CRS-R scores. The results of meta-analysis indicated that the CRS-R scores increased significantly as a result of tDCS when compared with the control group (*MD* = 1.28; 95% CI = 0.56–2.00; *P* < 0.01; Figure 7). Pooled studies were homogenous (*I*^2^ = 12%; *P* = 0.33). Moreover, on the basis of subgroup analysis for patient diagnoses, 11 studies reported the CRS-R scores of patients diagnosed with MCS, and five studies reported the CRS-R scores of patients diagnosed with UWS or VS (*MD* = −0.06; 95% CI = −0.56 to 0.43; *P* = 0.80; Figure 8). For patients with MCS, the results showed that the CRS-R scores increased significantly as a result of tDCS when compared with control group (*MD* = 1.65; 95% CI = 0.90–2.40; *P* < 0.01; Figure 8). The results of heterogeneity test showed that there was a significant heterogeneity across studies (*I*^2^ = 48%; *P* = 0.04). Therefore, the random-effects model was used for this subgroup data analyses (*MD* = 1.84; 95% CI = 0.74–2.93; *P* < 0.01). Furthermore, for the subgroup analysis of the stimulation doses, four studies conducted single session of tDCS (*MD* = 0.79; 95% CI = −0.41 to 1.98; *P* = 0.20; Figure 9), three studies conducted five sessions of tDCS totally (*MD* = 0.77; 95% CI = −0.46 to 2.00; *P* = 0.22; Figure 9), one study conducted ten sessions of tDCS totally (*MD* = 1.80; 95% CI = −3.31 to 6.91; *P* = 0.49; Figure 9). No heterogeneity was detected among these studies in above three subgroup analyses (*I*^2^ = 0%; *P* > 0.05). Moreover, four studies conducted more than 20 sessions of tDCS for patients with DOC (*MD* = 2.54; 95% CI = 1.15–3.92; *P* < 0.01). However, the result of heterogeneity test showed that there was a significant heterogeneity across studies in this subgroup analyses (*P* = 0.12; *I*^2^ = 49%), so the random-effects model was used for this subgroup data analyses (*MD* = 2.71; 95% CI = 0.58–4.84; *P* = 0.01).

## Discussion

Patients with DOC face a significant lack of treatment options, especially pharmacological ones, and therefore are unable to participate in active rehabilitation programs, which results in poor function outcomes. Neuromodulation techniques are alternative options to treat DOC. As a NIBS technique, tDCS can modulate cortical excitability by the direct current, but its therapeutic efficacy, especially behavioral effect, for DOC is not consistent. This systematic review, aimed to investigate the effect of tDCS for patients with DOC, included 17 eligible studies, and 15 studies with 580 DOC patients were included in the quantitative synthesis. The results of our meta-analysis showed that anodal tDCS can effectively enhance the recovery on GCS and CRS-R scores in patients with DOC.

Previous reviews summarized that patients with DOC could benefit from tDCS (Bourdillon et al., 2019; Thibaut et al., 2019b; Zaninotto et al., 2019), though the overall quality of evidence was not strong, which is consistent with our results. A recent systematic review and meta-analysis published by Feng et al. (2020) investigated the effect of NIBS for patients with DOC. The results of this study showed that anodal tDCS could significantly enhance the CRS-R scores in patients with DOC, which is also consistent with the results of our meta-analysis. Feng et al. (2020) stated that there is a lack of correlation between stimulation dose and effect sizes based on meta-regression, due to that behavioral changes may be too subtle to be detected by CRS-R in short-term tDCS. In our meta-analysis, however, we conducted subgroup analysis divided by total stimulation sessions and found that only more than 20 sessions of stimulation significant enhances the improvement of GCS scores and the CRS-R scores. Therefore, behavioral changes of patients with DOC require repetitive tDCS. Moreover, the different diagnosis of patients with DOC may be variously susceptible to tDCS intervention. The results of our meta-analysis showed that patients with DOC diagnosed with MCS were significantly benefit form tDCS on CRS-R scores improvement, while patients diagnosed with UWS or VS did not benefit, which is also in line with Feng’s results (Feng et al., 2020). The possible reasons are higher level of under-excitability of the DLPFC and lower capacity for neural plasticity in patients with UWS or VS (Monti, 2012). Bai et al. (2017) found that the global cerebral excitability increased in both MCS and VS patients after tDCS intervention, but the increased excitability of patients with VS in temporal and spatial domains was less than that of patients with MCS, which can partly explain why the behavioral changes of patients with VS are not as significant as those of patients with MCS.

The stimulation parameters of tDCS for patients with DOC, including electrode positioning, current intensity, stimulation duration, are without uniform standard. The brain targets of tDCS depends on the characteristics of anode electrode for modulating cortical excitability, and brain functional regions related to consciousness. Anodal or cathodal current could facilitate the depolarization or hyperpolarization of cortical neurons, respectively (Nitsche et al., 2003). The consciousness of human consists of two critical components: wakefulness and awareness (Steriade, 1996). Previous researches demonstrated that the wakefulness pathways originated in the brainstem activate awareness network and its thalamocortical network, which is conceptualized as the ascending reticular activating system (Parvizi and Damasio, 2001). Awareness is mediated by the brain cortex, which is superficial and therefore frequently chose as stimulating targets in NIBS researches (Zeman, 2006). The DLPFC is a key brain region to manage the higher cognitive functions which are closely related to awareness, and it is also found that stimulating DLPFC could release the inhibition of the thalamus which can facilitate the wakefulness (Thibaut et al., 2012). That is the reason why most NIBS studies chose DLPFC as brain target to promote consciousness recovery. Another brain target of tDCS is motor cortex, which was proved to be effective in promoting motor recovery for patients with neurological disorders (Lefaucheur et al., 2020). Therefore, some researchers thought behavioral changes measured by CRS-R could be detected by stimulating the motor cortex (Martens et al., 2019). The current intensity of all included studies was 1–2 mA which was thought a safety intensity for tDCS, and therefore no adverse events were reported in all included studies. However, current density is the main indicator to measure the safety of electrical stimulation, but few studies mentioned this concept in their stimulation protocols. It is also regrettable that no trials explored the relationship between stimulus intensity and the therapeutic effect for patients with DOC. The stimulation doses of included studies are various. The cortex excitability can be modulated by single session of tDCS, but no or only transient behavioral effects can be detected (Thibaut et al., 2014). What’s more, for the material of electrodes, one study used round rubber electrodes (12 cm^2^) (Barra et al., 2022), one study used eight gelled electrodes (3.14 cm^2^ Ag/AgCl) (Martens et al., 2020), and the rest studies all used saline-soaked surface sponge electrodes (35 cm^2^) (Thibaut et al., 2014, 2017, 2019a; Estraneo et al., 2017; Huang et al., 2017; Zhang et al., 2017, 2020; Chi et al., 2018; Martens et al., 2018, 2019; Cavinato et al., 2019; Wu et al., 2019; Chen et al., 2021; Guo et al., 2021; Li et al., 2021). Although the material of electrodes is related to the definition of tDCS, due to the limited number of studies, it is difficult to evaluate the therapeutic effect of different materials, and no studies has investigated the relationship of tDCS definition and therapeutic effect for patients with DOC. Physiologically, the establishment of the long-lasting after-effects depends on membrane potential changes as well as modulations of N-methyl-D-aspartic acid receptor efficacy, which can induce long-term potentiation and long-term depression-like effect (Cirillo et al., 2017; Kronberg et al., 2017; Kuo et al., 2017). Therefore, repeated tDCS is necessary for the long-term effect of DOC, which is consistent with our findings.

Consequently, based on the evidence provided by our study, tDCS is effective in promote DOC recovery, in terms of GCS scores and CRS-R scores. However, further researches regarding the mechanistic and optimal stimulation parameters of tDCS for DOC should be conducted.

### Study limitations

There are some limitations in our systematic review and meta-analysis. Firstly, studies published in languages other than English or Chinese were not included. Secondly, we only evaluated the behavior efficacy of tDCS for patients with DOC, and are unable to quantitatively analyses the neurophysiological changes due to the various methods of neuroimaging and neurophysiological assessments. Thirdly, because of the limited number of eligible studies and various of stimulation protocols, we are unable to recommend the optimal stimulation parameters. Fourthly, our results may be influenced by unavoidable heterogeneity as a result of that most studies did not strictly screen the patients for the onset time and diagnosis of DOC. Finally, outcomes of included studies were measured immediately after intervention without any long-term follow-up.

## Conclusion

In conclusion, the results of our studies indicated that anodal tDCS can effectively enhance the improvement in GCS and CRS-R scores in patients with DOC. Anodal tDCS with sufficient stimulation doses appears to facilitate recovery of consciousness for patients with MCS, in terms of CRS-R scores.

## Data availability statement

The raw data supporting the conclusions of this article will be made available by the authors, without undue reservation.

## Author contributions

HM, KZ, and CH: concept and idea, project management, and consultation. HM, KZ, and CJ: search design. HM and KZ: writing and data analysis. JY, MZ, and TW: data extraction and quality assessment. All authors contributed to the article and approved the submitted version.
