# Supplementary material for: Effect of transcranial direct current stimulation for patients with disorders of consciousness: A systematic review and meta-analysis
Source: Front Neurosci. 2023 Jan 23;16:1081278. doi: 10.3389/fnins.2022.1081278 (PMC9899861; doi:10.3389/fnins.2022.1081278)
Supplement: Supplementary file 2 [file Data_Sheet_2.docx]

MEDLINE and EMBASE via OVID

1. exp Transcranial Direct Current Stimulation/
2. exp Electric Stimulation Therapy/
3. exp Electric Stimulation/
4. exp Electrodes/
5. (transcranial adj5 direct current adj5 stimulation).tw.
6. (transcranial adj5 DC adj5 stimulation).tw.
7. (transcranial adj5 electric$ adj5 stimulation).tw.
8. (tDCS or A-tDCS or C-tDCS or S-tDCS or electrode$ or anode or anodes or anodal or cathode or cathodes or cathodal).tw.
9. 1 or 2 or 3 or 4 or 5 or 6 or 7 or 8
10. exp Coma/
11. exp Consciousness Disorders/ or exp Consciousness/
12. exp Persistent Vegetative State/
13. exp Glasgow Coma Scale/
14. exp Glasgow Outcome Scale/
15. exp Brain Injuries, Traumatic/ or exp Brain Injuries/
16. exp Brain Edema/
17. exp Craniocerebral Trauma/
18. exp Cerebrovascular Trauma/ or exp Cerebrovascular Disorders/
19. ((head or crani* or cerebr* or capitis or brain* or forebrain* or skull* or hemispher* or intra-cran* or inter-cran*) adj5 (injur* or trauma*or damag* or wound* or fracture* or contusion*)).tw.
20. ((head or crani* or cerebr* or brain* or intra-cran* or inter-cran*) adj5 (haematoma* or hematoma* or haemorrhag* or hemorrhag* orbleed* or pressure)).tw.
21. (Glasgow adj (coma or outcome) adj (scale* or score*)).tw.
22. Rancho Los Amigos Scale.tw.
23. Coma recovery scale.tw.
24. ((unconscious* or coma* or concuss* or 'persistent vegetative state') adj3 (injur* or trauma* or damag* or wound* or fracture*)).tw.
25. ("diffuse axonal injury" or "diffuse axonal injuries").tw.
26. ((brain or cerebral or intracranial) adj3 (oedema or edema or swell*)).tw.
27. 10 or 11 or 12 or 13 or 14 or 15 or 16 or 17 or 18 or 19 or 20 or 21 or 22 or 23 or 24 or 25 or 26
28. 9 and 27
29. exp Randomized Controlled Trial/
30. exp Random Allocation/
31. exp Controlled Clinical Trial/
32. exp Control Groups/
33. exp Clinical Trial/
34. exp Double-Blind Method/
35. exp Single-Blind Method/
36. exp Placebos/
37. exp Placebo Effect/
38. exp Cross-Over Studies/
39. ("randomized controlled trial" or "controlled clinical trial" or "clinical trial").pt.
40. (random$ or RCT or RCTs).tw.
41. (controlled adj5 (trial$ or stud$)).tw.
42. (cross-over or cross over or crossover).tw.
43. 29 or 30 or 31 or 32 or 33 or 34 or 35 or 36 or 37 or 38 or 39 or 40 or 41 or 42
44. exp animals/ not humans.sh.
45. 43 not 44
46. 28 and 45

Web of Science

1. TI=(Transcranial Direct Current Stimulation or Electric Stimulation Therapy or Electric Stimulation or Electrodes or tDCS or A-tDCS or C-tDCS or S-tDCS or electrode$ or anode or anodes or anodal or cathode or cathodes or cathodal)
2. AB=(Transcranial Direct Current Stimulation or Electric Stimulation Therapy or Electric Stimulation or Electrodes or tDCS or A-tDCS or C-tDCS or S-tDCS or electrode$ or anode or anodes or anodal or cathode or cathodes or cathodal)
3. 1 or 2
4. TI=(Coma or Consciousness Disorders or Consciousness or Persistent Vegetative State or Glasgow Coma Scale or Glasgow Coma Scale or Brain Injuries or Brain Edema or Craniocerebral Trauma or Cerebrovascular Trauma or Cerebrovascular Disorders or unconscious* or coma* or concuss* or Rancho Los Amigos Scale or Coma recovery scale)
5. AB=(Coma or Consciousness Disorders or Consciousness or Persistent Vegetative State or Glasgow Coma Scale or Glasgow Coma Scale or Brain Injuries or Brain Edema or Craniocerebral Trauma or Cerebrovascular Trauma or Cerebrovascular Disorders or unconscious* or coma* or concuss* or Rancho Los Amigos Scale or Coma recovery scale)
6. 4 or 5
7. 3 and 6
8. TI=(clinical or control* or placebo or random or randomised or randomized or randomly or random order or random sequence or random allocation or randomly allocated or at random or trial* or group* or study or studies or placebo or controlled)
9. AB=(clinical or control* or placebo or random or randomised or randomized or randomly or random order or random sequence or random allocation or randomly allocated or at random or trial* or group* or study or studies or placebo or controlled)
10. 8 or 9
11. 7 and 10

CENTRAL

#1MeSH descriptor: [Transcranial Direct Current Stimulation] explode all trees

#2 MeSH descriptor: [Electric Stimulation Therapy] explode all trees

#3 MeSH descriptor: [Electric Stimulation] explode all trees

#4 MeSH descriptor: [Electrodes] explode all trees

#5 tDCS or A-tDCS or C-tDCS or S-tDCS or electrode$ or anode or anodes or anodal or cathode or cathodes or cathodal

#6 (transcranial near/5 direct current near/5 stimulation) or (transcranial near/5 DC near/5 stimulation) or (transcranial near/5 electric$ near/5 stimulation)

#7 (#1 or #2 or #3 or #4 or #5 or #6)

#8 MeSH descriptor: [coma] explode all trees

#9 MeSH descriptor: [Consciousness Disorders] explode all trees

#10 MeSH descriptor: [Consciousness] explode all trees

#11 MeSH descriptor: [Persistent Vegetative State] explode all trees

#12 MeSH descriptor: [Glasgow Coma Scale] explode all trees

#13 MeSH descriptor: [Glasgow Outcome Scale] explode all trees

#14 MeSH descriptor: [Brain Injuries, Traumatic] explode all trees

#15 MeSH descriptor: [Brain Injuries] explode all trees

#16 MeSH descriptor: [Brain Edema] explode all trees

#17 MeSH descriptor: [Craniocerebral Trauma] explode all trees

#18 MeSH descriptor: [Cerebrovascular Trauma] explode all trees

#19 MeSH descriptor: [Cerebrovascular Disorders] explode all trees

#20 (head or crani* or cerebr* or capitis or brain* or forebrain* or skull* or hemispher* or intra-cran* or inter-cran*) near/5 (injur* or trauma*or damag* or wound* or fracture* or contusion*)

#21 Rancho Los Amigos Scale or Coma recovery scale

#22 (unconscious* or coma* or concuss* or 'persistent vegetative state') near/3 (injur* or trauma* or damag* or wound* or fracture*)

#23 (#8 or #9 or #10 or #11 or #12 or #13 or #14 or #15 or #16 or #17 or #18 or #19 or #20 or #21 or #22)

#24 (#7 and #23) limited in trials

Physiotherapy evidence database (PEDro)

Abstract and Title: transcranial direct current stimulation

Method: Clinical trial

All search terms in the title or abstract were combined with method descriptors using AND operator.

CNKI, Weipu and Wang Fang

1. topic (经颅直流电刺激) or (tDCS)
2. topic (昏迷) or (意识障碍) or (植物状态)
3. 1 and 2
